# Transcriptome Bioinformatical Analysis of Vertebrate Stages of *Schistosoma japonicum* Reveals Alternative Splicing Events

**DOI:** 10.1371/journal.pone.0138470

**Published:** 2015-09-25

**Authors:** Xinye Wang, Xindong Xu, Xingyu Lu, Yuanbin Zhang, Weiqing Pan

**Affiliations:** 1 Institute for Infectious Diseases and Vaccine Development, Tongji University School of Medicine, Shanghai, China; 2 School of Life Science and Biotechnology, Shanghai Jiao Tong University, Shanghai, China; 3 Department of Tropical Infectious Diseases, Second Military Medical University, Shanghai, China; National Institute of Health—National Cancer Institute, UNITED STATES

## Abstract

Alternative splicing is a molecular process that contributes greatly to the diversification of proteome and to gene functions. Understanding the mechanisms of stage-specific alternative splicing can provide a better understanding of the development of eukaryotes and the functions of different genes. *Schistosoma japonicum* is an infectious blood-dwelling trematode with a complex lifecycle that causes the tropical disease schistosomiasis. In this study, we analyzed the transcriptome of *Schistosoma japonicum* to discover alternative splicing events in this parasite, by applying RNA-seq to cDNA library of adults and schistosomula. Results were validated by RT-PCR and sequencing. We found 11,623 alternative splicing events among 7,099 protein encoding genes and average proportion of alternative splicing events per gene was 42.14%. We showed that exon skip is the most common type of alternative splicing events as found in high eukaryotes, whereas intron retention is the least common alternative splicing type. According to intron boundary analysis, the parasite possesses same intron boundaries as other organisms, namely the classic “GT-AG” rule. And in alternative spliced introns or exons, this rule is less strict. And we have attempted to detect alternative splicing events in genes encoding proteins with signal peptides and transmembrane helices, suggesting that alternative splicing could change subcellular locations of specific gene products. Our results indicate that alternative splicing is prevalent in this parasitic worm, and that the worm is close to its hosts. The revealed secretome involved in alternative splicing implies new perspective into understanding interaction between the parasite and its host.

## Introduction

*Schistosoma japonicum*(*S*. *japonicum*) is an infectious blood-dwelling trematode that causes schistosomiasis in numerous mammals, including humans. This chronic disease affects more than 200 million people in tropical and subtropical regions and leads to high morbidity and mortality [1, 2]. *S*. *japonicum* has a complicated lifecycle involving an intermediate snail host and a definitive mammalian host in which it resides in the hepatic portal and mesenteric veins. Females produce numerous eggs that can be transported via portal veins to the liver that elicit a severe immune response from the host [3]. The parasite has adopted sophisticated strategies to adapt to contrasting environments of different hosts and to evade the pressure of host immune system, yet these remain poorly understood.

Alternative splicing (AS) is a transcriptional process of pre-mRNAs that enables one gene to encode two or more mature mRNAs [4]. AS could explain exactly why a limited number of genes could produce a vast number of proteins [4, 5]. AS is widespread in eukaryotes, and it has been estimated that >95% of human genes are alternative spliced [6]. Despite its importance, AS is not well-characterized.

Next-generation sequencing has enabled analyses of genomes and transcriptomes with unprecedented coverage and depth, revealing global AS events in transcriptomes of many animals [7]. Plants, cows, mice and invertebrates such as *Caenorhabditis elegans* has been delineated landscapes of alternative splicing events in genome scales using next-generation sequencing[8–13]. The genomes of *S*. *japonicum* and *S*. *mansoni* were sequenced and assembled in 2009 [14, 15]. Further studies using this genomic data have uncovered many instances of AS of protein encoding genes in *S*. *mansoni* [16–19], including the MEG gene family [20]. And recently in *S*. *japonicum*, there was one research investigating AS events in female and male worm and revealed lots of AS events [21]. In this study, we analyzed the transcriptome of *S*. *japonicum* to discover AS events in vertebrate stages of this parasite, by applying RNA-seq to cDNA library of adults and schistosomula. Our data could be complementary to further re-annotate transcriptome of the parasite.

## Materials and Methods

### Ethics Statement

Ethical approval for the study was received from the Institutional Animal Care and Use Committee of Tongji University, with the pemit number: TJMED-011-041. All animal experiments were performed in accordance with the Regulations for the Administration of Affairs Concerning Experimental Animals approved by the State Council of People’s Republic of China.

### Parasites and animals

**Fig 1:**
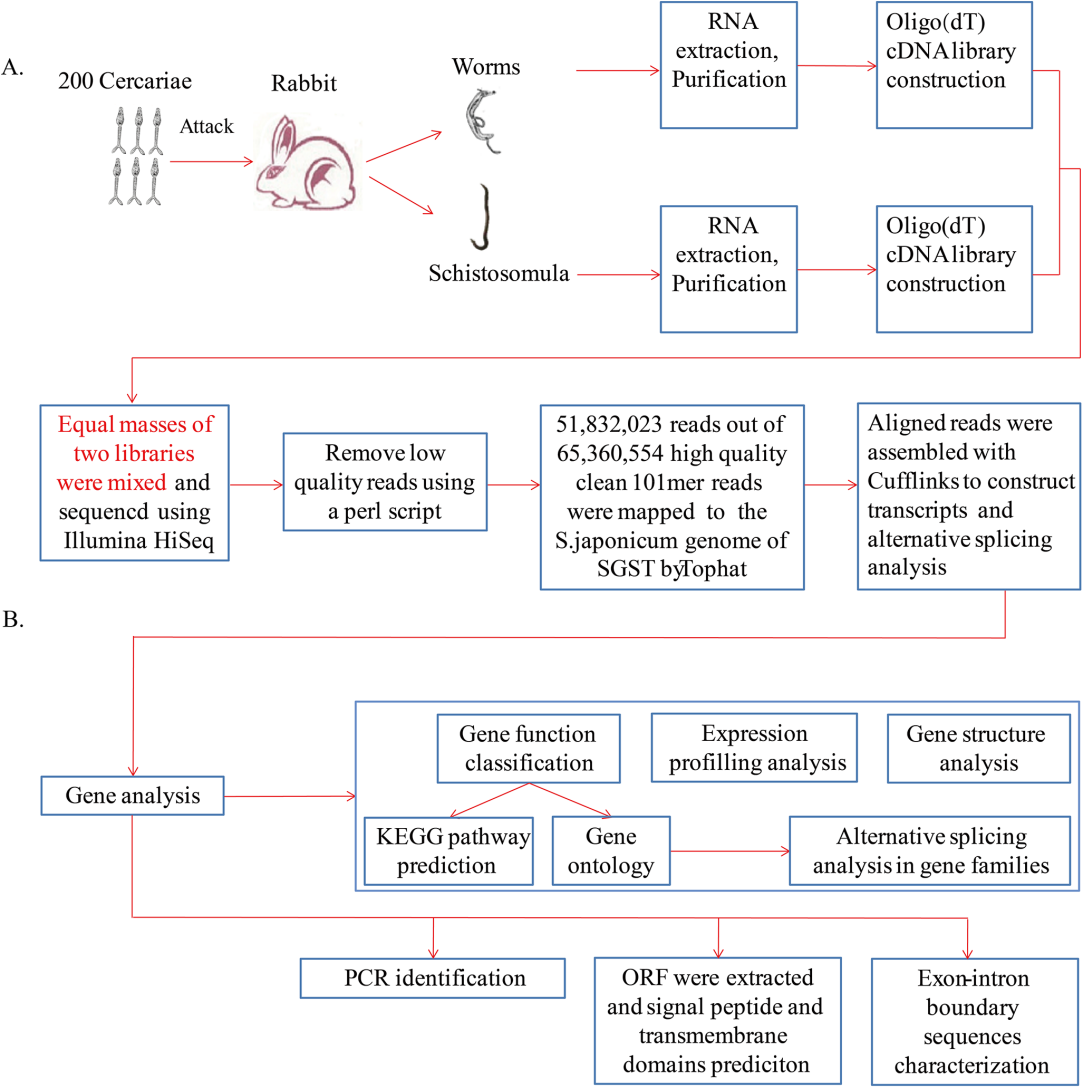
Outlines of RNA-seq and data analysis for identifing AS in *Schistosoma japonicum*. A, Design and material collection for RNA-seq. The library sequenced here was mixture of equal amount cDNA of adult worms and schistosomula. B, Computational data analysis and experimental idenfication.

Cercariae were provided by the National Institute of Parasitic Disease, Chinese Center for Disease Control and Prevetion (CDC), Shanghai, and collected using a light induction method [22]. From these, we obtained schistosomula and adult worms by vein perfusion of Balb/c mice (bought from Slac laboratory animal Co.td, bred in clean grade) infected with ~40 cercariae each on day 21 or day 42 post-infection. One New Zealand white female rabbit (bought from Slac laboratory animal Co.td, bred in clean grade) was infected with 200 cercariae and then euthanized on day 42 post-infection. Eggs of *S*. *japonicum* were harvested from the infected rabbit liver according to the protocol of Ashton et al. [23] with some modifications. Briefly, livers were cut into pieces and mechanically homogenized in a Waring blender in 1X PBS. The homogenate was centrifuged at 3500 rpm at 4°C for 5 min and the sediment was rinsed three times with 1X PBS. Then the sediment was suspended in Hank’s solution with 1 mg/mL collagenase IV and was incubated at 37°C at 250 rpm overnight. The digested homogenate was then spun again and rinsed three times until the supernatant was clean. Then pellet was re-suspended in 1X PBS and added into a column with an 8 mL Percoll (Gibco) and 32 mL 0.25M sucrose solution. Then column was centrifuged at 4°C at 800 g for 10 min. The supernatant was discarded and the pellet was washed with 1X PBS containing 1mM EDTA and 1 mM EGTA. Then EDTA and EGTA were washed using above Percoll column. The pellet was washed with 1X PBS until clean. All the worms and eggs were stored in TRIzol reagent (Thermo Scientific). A schematic diagram of the methodological steps taken in this study is shown in Fig 1.

### RNA isolation and cDNA library construction

Total RNA was isolated using TRIzol reagent (Thermo Scientific), following by RNA purification by RNeasy MiniElute Cleanup Kit (Qiagen), according to the manufacturer’s instructions. For library construction, mRNA was purified from total RNA using poly-T oligo-attached magnetic beads (Illumina) (Fig 1). The purified mRNA was cut into fragments with an average length of 155 bp using divalent cations at 95°C for 8 min. The cleaved mRNA fragments were reverse-transcribed into first strand of cDNA using random hexamers, prior to the synthesis of the second strand of cDNA. After end repair process, cDNA fragments were linked with adaptors and amplified by PCR.

### RNA-sequencing and assembly

Equal amount of cDNA libraries of schistosomula and adults were mixed and the final concentration of this mixed library was adjusted to 2 nM (Fig 1). The sample was then sequenced on an Illumina HiSeq system. All reads were qualified and a custom Perl script was written to remove low-quality reads (those where more than half bases had a quality value <5). The retained high quality reads were mapped to the *S*. *japonicum* genome of SGST (http://lifecenter.sgst.cn/) by Tophat (version v2.04) [24] and then assembled by Cufflinks (Version 2.00) [25] to construct unique transcripts sequences and AS analysis, using the parameter:-g-b-u-o. All sequencing data could be obtained in NCBI with accession number GSE71722.

### Functional annotation and classification

Unigenes were first compared with the Kyoto Encyclopedia of Genes and Genomes database (KEGG, release 58) using BLASTX at E values ≤1e^-10^. A custom Perl script was written to retrieve KO information from the blast result and then established pathway associations between unigenes and the database (Fig 1). Interpro domains were annotated by InterProScan Release 27.0 (http://www.ebi.ac.uk/interpro/) and functional assignments were mapped onto Gene Ontology (GO). WEGO was used to do GO classification and draw GO tree.

As secreted proteins, including transmembrane proteins, play a pivotal role in communication between parasites and their hosts, we focused on inspecting alternative splicing events among secretory and excretory protein encoding genes. For open reading frames (ORFs) predictions, alternative splicing genes were extracted from total genes and then open reading frames were predicted using online prediction tool emboss explorer (http://emboss.bioinformatics.nl/) using standard code. The repeated ORFs of one transcript were checked and removed, and only the longest ORF was kept for the transcript. The ORFs were translated into corresponding amino acid sequences also using Emboss explorer, defined as translating in the 1st frame, using standard codes. Afterwards, AS amino acid sequences were processed using online prediction tools SignalP and TMHMM to find potential signal peptides and transmembrane helices among these transcripts. SignalP 4.1 server was used with D-cutoff values set as Default.

### PCR validation

To eliminate contamination by genomic DNA, all isolated RNAs were treated with DNase I (Thermo Scientific) and then RNAs were extracted by phenol-chloroform. The total RNAs was then reverse-transcribed using random hexamers and poly T hexamers (TaKaRa). The cDNAs of four life stages (including adult worms, schistosomula, cercariae and eggs) of *S*. *japonicum* were used as PCR templates.

To validate alternative splicing genes experimentally, RT-PCR were performed for 45 randomly selected genes in four life stages of *Schistosoma japonicum*. Primers were designed by following principle: one of the paired primers crossed the boundary of the skipped exon or retained intron and neighboring cassette exon, and the other was designed to another constitutive exon, thus alternative variants could be amplified. And primers annealing to constitutive exons were also designed to monitor whether two different isoforms were expressed in individual life stage.

PCR was performed using cDNA templates of four stages of the parasite and two pairs of primers. PCR was carried out using rTaq (TAKARA) and the protocol as follows: initial denaturation at 95°C for 5min, then 95°C for 30 s, 55°C for 30 s and 72°C for 30 s for 35 cycles, and a final extension in 72°C for 10 min. The PCR products were visualized on 1.5% agarose gels.

## Results

### Analysis of RNA-seq in *S*. *japonicum*

**Fig 2:**
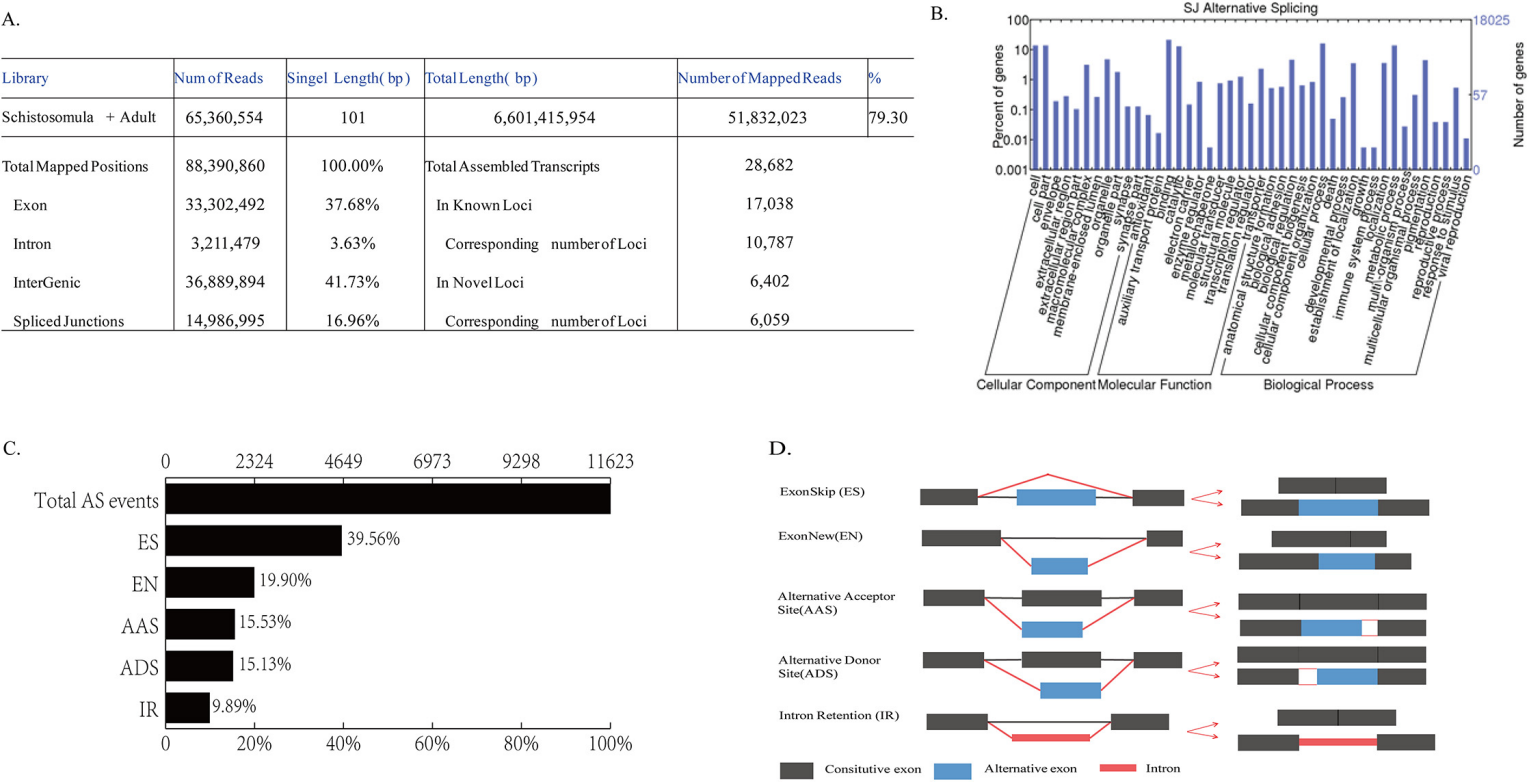
Analysis of RNA-seq data and alternative splicing in *Schistosoma japonicum*. A, Reads statistics in sheet form. In the mixed library, 65,360,554 reads with average length of 101 bp were detected. 79.30% of them were aligned with genome of *Schistosoma japonicum*. B, Gene Ontoloy annotation of alternative spliced genes in *Schistosoma japonicum*. C, Alternative splicing discovered in our data. ES: exon skip; EN: exon new; AAS: alternative acceptor site; ADS: alternative donor site; IR: intron retention. D, Annotation of five alternative splicing types in sketch.

In total we obtained 65,360,554 high quality reads with a total length of 6,601,415,954 bp. These high quality reads were mapped to *S*. *japonicum* genome of SGST by same Tophat version (v 2.04) for two independent mapping analyses [24] (Fig 2A, S1 Table). In the first mapping, 79.3% (51,832,023 reads) were mapped to the genome while 78.12% (51,057,431 reads) were mapped for the second mapping. All the subsequent assemblies of reads and analyses such as gene annotation and alternative splicing were based on the first mapping results.

We identified a total of 18,025 genes in the parasite (Table 1, S2 Table), with 16,846 of these expressed in adult and schistosomulum life stages. We observed 11,623 AS events in 7,099 genes in these two life stages. The average proportion of AS events per gene was 42.14%.

**Table 1:** Statistics of genes indentified in RNA-seq data.

Total genes	18025
Total assembled transcripts	28682
Expressed genes^a^	16846
AS events	11623
Number of genes occur AS	7099
AS percentage^a^	42.14%

^a^ Genes that expressed and alternative spliced in the samples of adult worms and schistosomula.

All AS events were classified into five groups: exon skip (4,598), intron retention (1,149), alternative donor site (1,758), alternative acceptor site (1,805) and exon new (2,313). New exons consisted of exons from our sequencing data that were missed in reference database (http://lifecenter.sgst.cn/schistosoma/en/schdownload.do). In addition, some of our sequences were longer than reference sequences in 5’ or 3’ end (or both), in what we term 3’-extension (6,280) or 5’-extension (4,791), perhaps because the annotation of reference genome was incomplete, and hence, some AS events were not counted in the final AS statistics. We found that exon skip (ES) was the most common form of splicing, whereas intron retention (9.89%) was the least common splicing form (Fig 2B). Our analysis revealed that non-specific types of genes underwent more AS events than did other gene types, such as some house-keeping genes (those involved in growth and the metallo-chaperone; Fig 2D).

### Gene families involved in alternative splicing events

Gene ontology annotation enabled functions of genes to be predicted and we analyzed seven of the gene families presented in *S*. *japonicum* (Table 2). The proportion of AS events in many gene families was similar to average AS rate of 42.14% (e.g. solute carrier family, signal pathways in WNT family, Notch gene family and Ras pathway related genes; Table 2). However, AS rates for switch/sucrose non-fermentable (SWI/SNF) family and transforming growth factor (TGF-β) family was much higher than average, while AS was rare in G-protein coupled receptors (GPCR) family.

**Table 2:** Statistics of alternative splicing in different gene families.

Gene family	Member number	Alternative spliced	Percentage
GPCR family	71	12	16.90%
WNT family	71	42	59.20%
SWI/SNF related	13	10	76.90%
Ras related	36	18	50%
Notch pathway related	28	18	64.30%
Solute carrier family	114	55	48.20%
TGF-β family	33	24	72.70%

### PCR verification of alternative variants in four life stages of *S*. *japonicum*

To confirm alternative splicing genes experimentally, RT-PCR were performed among 45 randomly selected genes (Tables 3 and 4, S3 Table). We selected four life stages of *Schistosoma japonicum*, which were most related to its mammalian hosts, for PCR identification: adult stage, schistosomulum stage, cercariae stage and egg stage. Among genes identified, it’s not common that two different transcript variants of one gene exist simultaneously in one life stage. As shown in Tables 3 and 4, many of the expressed genes had one isoform per life-cycle stage. It was also evident that many genes were turned off in cercariae and egg stages (Tables 3 and 4).

**Table 3:** Summary of identified alternative spliced genes (I).

Gene ID	Life Stages								GO Annotation
	Adu^a^		Sch^a^		Cer^a^		Egg^a^		
	A^b^	B^c^	A^b^	B^c^	A^b^	B^c^	A^b^	B^c^	
CUFF.37	-	+	-	+	-	+	-	+	DEAD box ATP-dependent RNA helicase
CUFF.250	N.A		+	+	+	+	+	+	mitochondrial carrier protein
CUFF.937	+	-	+	-	+	-	+	-	heterogeneous nuclear ribonucleoprotein K
CUFF.1080	+	-	+	-	N.A		+	-	hypothetical protein
CUFF.2046	+	-	+	-	+	-	-	-	hypothetical protein
CUFF.1255	+	-	+	-	+	-	+	-	hypothetical protein
CUFF.7161	+	-	N.A		N.A		N.A		*
CUFF.1666	+	+	+	+	+	+	+	+	hypothetical protein
CUFF.1653	+	+	+	+	+	-	N.A		Rac guanyl-nucleotide exchange factor
CUFF.1311	+	+	+	+	N.A		N.A		hypothetical protein
CUFF.1674	+	-	+	-	+	-	N.A		anaphase-promoting complex subunit 4
CUFF.1700	+	-	+	-	N.A		N.A		hypothetical protein
CUFF.1727	+	-	+	-	+	-	N.A		paramyosin
CUFF.1778	+	-	-	-	+	-	N.A		adenylosuccinate synthetase
CUFF.1871	+	-	+	-	N.A		N.A		phospholipase DDHD1
CUFF.1889	+	-	+	-	N.A		+	-	elav (embryonic lethal abnormal vision drosophila)-like protein
CUFF.1912	-	+	-	+	N.A		-	+	hypothetical protein
CUFF.1995	+	+	+	+	N.A		+	-	hypothetical protein
CUFF.2013	+	+	+	+	N.A		+	-	rex4-related (xpmc2)
CUFF.2149	-	+	-	+	N.A		-	+	septin
CUFF.2274	+	-	+	-	+	-	+	-	*
CUFF.2294	+	-	+	-	N.A		N.A		subfamily T1A non-peptidase homologue; 20S proteasome subunit alpha 5
CUFF.2331	+	-	N.A		N.A		N.A		3-hydroxy-2-methylbutyryl-CoA dehydrogenase
CUFF.2337	-	+	-	+	N.A		N.A		growth hormone secretagogue receptor
CUFF.3103	-	+	-	+	N.A		N.A		calcineurin B subunit; protein phosphatase 3, regulatory subunit

^a^ Adu: adult stage; Sch: schistosomulum stage; Cer: cercariae stage; Egg: egg stage.

^b^ means PCR product which contains alternative exon or intron.

^c^ means PCR product which doesn’t contain alternative exon or intron.

+: means correspoding band exists.

-: means correspoding band doesn’t exist.

N.A: means in the given life stage, neither of the isoforms were amplified.

*: means according to GO annotation, no specific function was annotated for the gene.

**Table 4:** Summary of identified alternative spliced genes (II).

Gene ID	Life Stages								GO Annotation
	Adu^a^		Sch^a^		Cer^a^		Egg^a^		
	A^b^	B^c^	A^b^	B^c^	A^b^	B^c^	A^b^	B^c^	
CUFF.3457	+	+	+	+	N.A		N.A		Smad4; SMAD, mothers against DPP 4
CUFF.5359	+	-	+	-	N.A		N.A		dishevelled protein
CUFF.5727	-	+	-	+	N.A		-	+	casein kinase II beta subunit
CUFF.11194	N.A		-	+	N.A		-	+	jnk/sapk-associated protein
CUFF.11604	+	+	+	+	N.A		+	+	protein phosphatase 2C
CUFF.11720	+	+	+	+	N.A		+	+	sodium-dependent neurotransmitter transporter
CUFF.11805	+	+	+	+	N.A		N.A		erythrocyte membrane protein
CUFF.11822	+	-	+	-	N.A		N.A		phospholipase D
CUFF.11899	+	+	+	+	N.A		+	+	ubiquitin carboxyl-terminal hydrolase 12/46
CUFF.11984	+	+	+	+	N.A		N.A		Bardet-Biedl syndrome 5
CUFF.12732	+	+	+	+	-	+	+	+	survival motor neuron protein; survival of motor neuron-related-splicing factor 30
CUFF.12753	+	-	+	-	N.A		+	-	ubiquitin-protein ligase mind-bomb
CUFF.12798	-	+	-	+	N.A		-	+	hypothetical protein
CUFF.12885	+	+	+	+	-	+	+	+	*
CUFF.12947	+	-	+	-	N.A		+	-	Slc9a7 protein
CUFF.13574	+	-	+	-	N.A		N.A		hypothetical protein
CUFF.13909	+	-	+	-	N.A		N.A		cadherin
CUFF.13989	-	+	-	+	N.A		N.A		hypothetical protein
CUFF.14192	N.A		+	-	N.A		+	+	*
CUFF.14312	N.A		+	-	N.A		N.A		hypothetical protein

^a^ Adu: adult stage; Sch: schistosomulum stage; Cer: cercariae stage; Egg: egg stage.

^b^ means PCR product which contains alternative exon or intron.

^c^ means PCR product which doesn’t contain alternative exon or intron.

+: means correspoding band exists.

-: means correspoding band doesn’t exist.

N.A: means in the given life stage, neither of the isoforms were amplified.

*: means according to GO annotation, no specific function was annotated for the gene.

**Fig 3:**
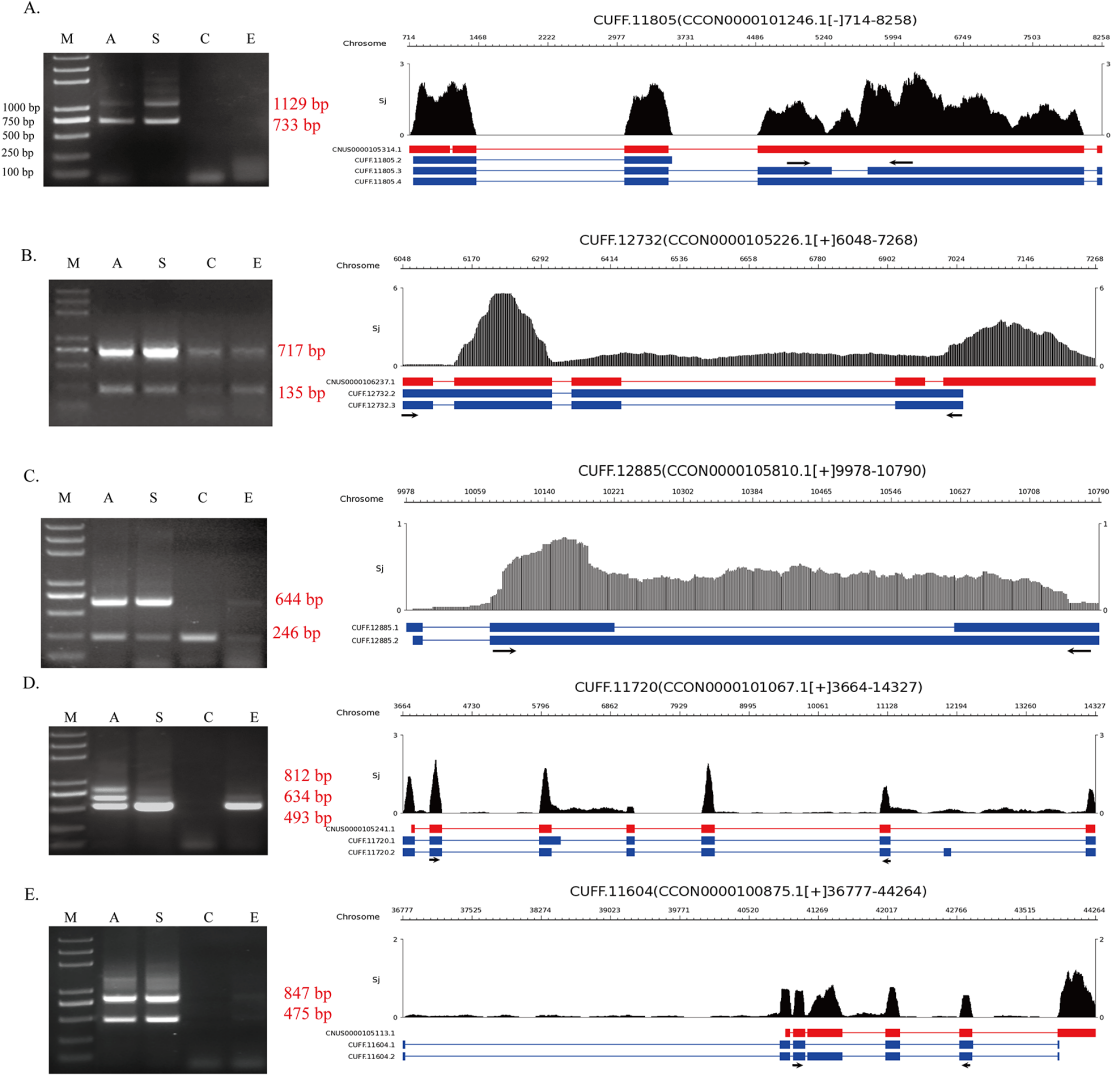
PCR identification of alternative spliced genes. Five examples are shown in the figure that represent different kinds of AS events: A and B, intron retention, C, new transcripts, D, alternative donor site, E, exon skip. PCR products of cDNAs of four life stages of *S*.*japonicum* were monitored by 1.5% agrose gel electrophoresis. Marker size was shown in first gel photo on left. Predicted products lengths are indicated in red font in right side of each gel photo, and arrows in the gene sketchs indicates where primers were designed. M: Marker; A: Adults; S: Schistosomula; C: Cercariae; E: eggs.

Here we present examples of five predicted genes that were verified by PCR (Fig 3). Gene CUFF.11805 (Reference gene access number: CNUS0000105314.1) and CUFF.12732 (Reference gene access number: CNUS0000106237.1) (Fig 3A and 3B) were both intron retained, CUFF.11805 encodes erythrocyte membrane protein, and seems to express specifically in adult and schistosomulum. CUFF.12732 encodes survival motor neuron protein, and the “short” transcript is expressed in all four stages, whereas the “longer” one doesn’t exsit in cercaria. Gene CUFF.12885 (No reference mRNA was found in the database, which means it’s probably a new gene.) seems to be expressed in a very low level in egg stage as two bands were slim, and in both adult and schistosomulum, two transcripts variants were amplified (Fig 3C). CUFF.11720 undergoes another alternative splicing pattern: alternative donor site (Fig 3D). It encodes sodium-dependent neurotransmitter transporter. Nevertheless, three different transcript variants were amplified in adult, which means this gene may experienced two or more alternative splicing. CUFF.11604 represents exon skip, and it’s inferred that this gene is only turned off in cercariae (Fig 3E). But alternative splicing of CUFF.11604 is not stage-specific.

### Alternative splicing motifs

**Fig 4:**
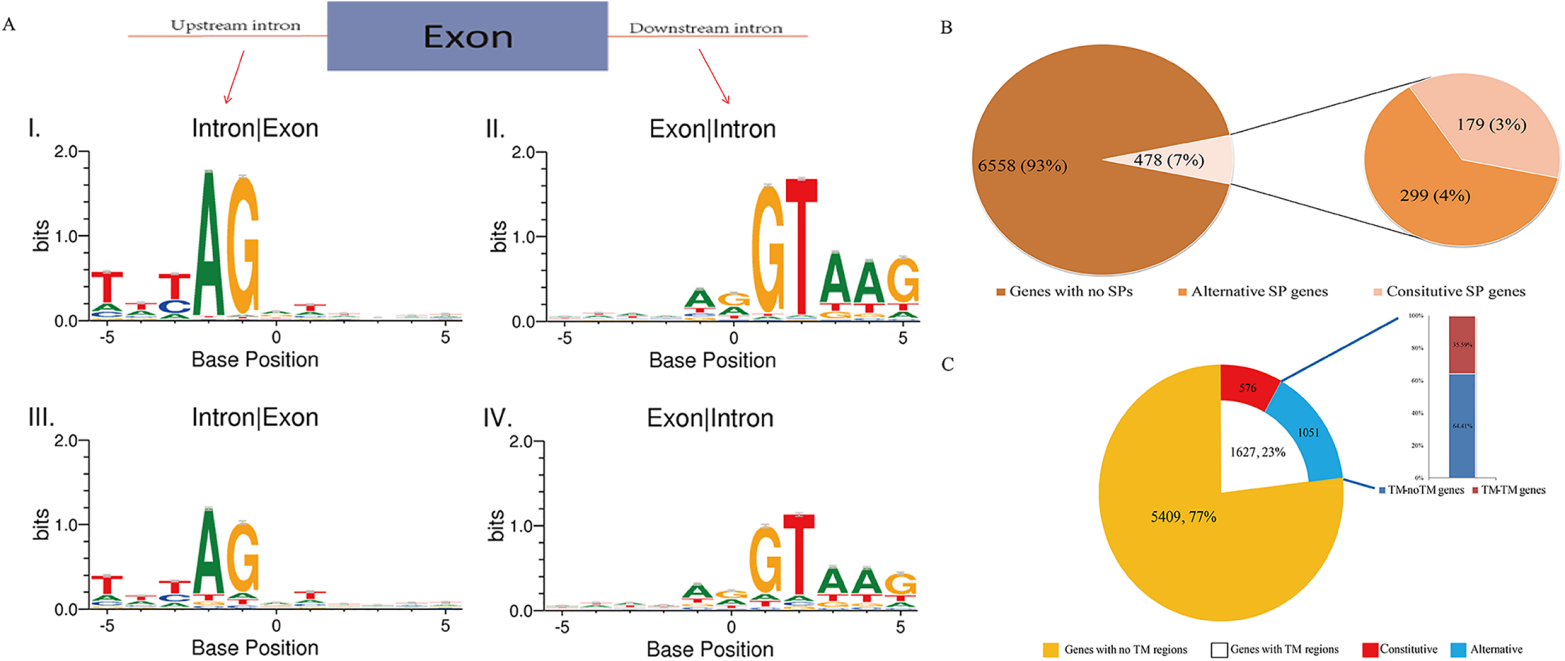
Binding motif search and alternative splicing in excretory and secretory proteins encoding genes. A, Sequence situations in boundaries of Cassette exons and alternative exons. Four bases frequencies in both ends of an exon and flank introns in five positions were calculated and showed by Weblogo 3.3 (http://weblogo.threeplusone.com/). I, left boundaries of cassette exons, namely intron-exon boundaries. II, right boundaries of cassette exons, namely exon-intron boundaries. III, left boundaries of alternative exons. IV, right boundaries of cassette exons. B, Alternative splicing in genes encoding proteins with signal peptides (SPs). Signal peptides were predicted using online tool SignalP 4.1 server, setting as default (D-cutoff for SignalP-noTM networks is 0.45 and D-cutoff for SignalP-TM networks is 0.5). Numbers in the pie chart imply gene amounts and percentages. Genes with no SPs: genes predicted as encoding transcripts with no signal peptides. Alternative SP genes: genes predicted as encoding multiple transcripts with or without signal peptides. Constitutive SP genes: genes predicted as encoding multiple transcripts with signal peptides. C, Alternative splicing in genes encoding proteins with transmembrane (TM) domains. Transmembrane domains were predicted using online tool TMHMM server2.0, setting as default. Annotations in the pie chart indicate gene amount and percentage. Constitutive: genes encode transcripts possessing transmembrane domains without alternative splicing found. Alternative: genes encode transcripts possessing transmembrane domains with alternative splicing found; TM- noTM genes: genes encode multiple transcripts with and without TM domains; TM-TM genes: genes encode multiple transcripts possessing different amount of TM domains.

We did not identify statistically significant motifs in alternative exons. However, we found that introns flanking both alternative and constitutive exons exhibited boundaries that appeared same to those found in other higher eukaryotes (Fig 4A). Moreover, in constitutive exons, splice sites are stricter than those in alternative exons. GT accounts for 92.3% of right boundaries in cassette exons, whereas 78.6% in alternative exons. And AG accounts for 94.4% of left boundaries in cassette exons, whereas 80.3% in alternative exons. In 3’ site of introns, trimerical T seems to be more prevalent in front of AG boundary than other bases combinations.

It is universally accepted that in higher eukaryotes intron boundaries obey the GT-AG rule, namely a GT at the 5’ end of introns and an AG at the 3’ end of introns is most common in numerous species, with a very low proportion of GC-AG, and AU-AC even rarer [26, 27]. The more complex the organism is, the more conserved this rule is. Interestingly, our findings of the proportion of base compositions in the introns in *S*. *japonicum*, conform to this rule. Generally the lower an organism is, the fewer introns it has and stronger the splice site is, resulting in few or no AS events [28]. Our results showing that *S*. *japonicum* has many AS events in both adults and schistosomula stages with common intron boundary rules, suggests close relationships between the parasite and its mammal hosts.

### Alternative splicing in the genes of secreted proteins

In our study we reported that of the 7,036 AS genes chosen, transcripts and transcripts variants of 478 (6.79%) genes were predicted with signal peptides (Fig 4B). Among these 478 genes, 299 were found to generate one or more transcripts without signal peptides. The remaining 179 AS genes encoded constitutive signal peptides in different transcripts variants.

As for transmembrane domains predictions, TMHMM server v.2.0 was used following instructions showed on the webpage. We found that of the 7,036 AS genes manipulated, 23.12% (1,627) were transcribed into at least one transcript with one or more transmembrane helices (Fig 4C). 576 of these genes (8.19%) encoding different transcript variants with no changes in transmembrane helices amounts, suggesting that AS does not change transmembrane domains in these 576 genes. For 374 of remaining 1,051 genes, we observed that they encoded transmembrane transcripts with different amount of transmembrane domains, while the other 677 genes encoded various transcripts, of which at least one transcript variant possessed no transmembrane domains.

These results suggested that AS may occur in transmembrane domain-encoding regions and function to change the amounts of transmembrane domains or alter it into non-transmembrane proteins. Together with SignalP prediction results (Fig 4B), these data suggested that AS may not only regulate gene activities and expression profiles, but also alter gene transcripts into functional, temporal and spatially diverse proteins to optimize the employment of genes and related regulatory factors.

## Discussion

Gene functions in forms of different proteins in different physiological progresses and environments, and this course could be modulated by transcriptional modifications, such as alternative splicing. Despite its importance, AS has only received research attention since the completion of human genome project. Different combinations of cis-acting elements and trans-acting factors regulate gene transcription and splicing to produce different transcripts. Also, regulations of gene transcription and splicing can span different genes, forming a sophisticated regulation network [29, 30]. Although several splicing factors and their subtypes have been identified [31–35], further investigation is needed to depict the whole landscape of alternative splicing.

*S*. *japonicum* is an important parasitic metazoan with a complex lifecycle that involves exposure to multiple environments. Despite significant advances in mapping its genome, few studies have investigated AS in this species, though Piao et al. recently reported AS events in male and female *S*. *japonicum* parasite [21]. Differing from Piao’s report, this study revealed transcripts and AS events in the adult worms and schistosmula. Consequently, over two thousands of unique novel genes and over one thousand of unique known genes were identified from this study in *S*. *japonicum*. In addition, we performed extra analysis including analysis of alternative splicing events in various gene families, alternative splicing motifs, and alternative splicing events in secretory protein- and transmembrane protein-encoding proteins. Nevertheless, PCR verification of alternative variants was carried in four life stages of the parasite.

We reported a total of 18,025 predicted genes whereas Piao’s study illustrated 15,939 and 19,501 predicted genes in female and male worms, respectively. We further compared predicted genes from two studies and found differences in the number of known and novel genes (S4 Table). A total of 10,834 known genes predicted in both studies were identical while 68 genes were unique in Piao’s study and 1,132 unique genes in this study (S1A Fig, S5 and S6 Tables). In all novel genes, 3,978 genes predicted in both studies were overlapped, while 5,308 genes were unique in Piao’s study and 2,081 genes unique in our study (S1B Fig, S7 and S8 Tables). These unique genes in our study may be expressed specificly in schistosomula stage and since in Piao’s study, it failed to detect these unique genes. This finding provided additional predicted genes including these novel genes that strongly supported re-annotating the genomic sequences of the parasite.

In terms of alternative splicing annotation, total alternative splicing events in female and male worms are 13,438 and 16,507 respectively, according to Piao’s report. And we identified 11,623 alternative splicing events in the transcriptomes of adults and schistosomula, with AS rates as high as 42.14%. We showed that the most common type of AS events was exon skip, while intron retention (IR) was the least common type of AS events [8]. In plants, it has been reported that intron retention is the most prevalent form of splicing, while exon skip is the most common form in higher eukaryotes [8]. Hence, our results suggest that *S*. *japonicum* is closer to animals. This is different from the findings in Piao’s study which showed that Alternative donor site (ADS) and Alternative acceptor sites (AAS) were more common than other two alternative splicing types. The difference of prevalent alterative splicing types between two studies could be due to different analysis and algorithms. In addition, the different RNA-seq samples prepared from two studies may also contribute to this discrepancy. Actually both studies contained 5’-extended and 3’-extended transcripts because of discontinuity and incomplete annotation of reference database. We excluded those ADS and AAS events when they occurred in exons located in 5’-extension or 3’-extension, as we have calculated that in 5-extension and 3’-extension, ADS and AAS counted for over 90%. We considered that these events were more likely to be false positive alternative splicing events because of imperfect annotation of genome of *S*. *japonicum*.

Our estimate of AS events could be underestimated because *S*. *japonicum* experiences several distinct environments during its complex life-cycle, yet we only sequenced two of four life stages related to mammal hosts, and more importantly, utilized an imperfect database as reference. Thus, some of our sequenced genes may be missing from reference databases. Another confounding issue is that *S*. *japonicum* genome was established solely from adult worms, and may not reflect genetic variation in other life stages [15]. However, given that *S*. *japonicum* lives in a variety of environments, many genes are likely to be subject to sophisticated regulation in different life stages. In this study, we found 18,025 genes in the genome, while 3,907 genes didn’t transcribe into mRNAs in adult and schistosomulum stage. This indicates that these genes might be active in eggs or other life stages, necessitating the synthesis of stage-specific gene expression.

Importantly, we also discovered novel transcripts and verified disparate rates of AS among different gene families of *S*. *japonicum*, likely explained by their different functions. GPCRs are famous biological receptors anchored in plasma membrane with a seven central transmembrane helix domains. Many studies provided evidences that many GPCRs commonly function as monomers, and some as heterodimers, homodimers, or higher structure oligomers [36, 37]. In eukaryotes, GPCRs function in sensing a broad range of extracellular stimuli, including photons, ions, peptides, proteins and other small molecules. It has been reported that in human genome, approximately 50% of GPCR genes lack introns, with those comprised of multi-exons exhibiting extensive alternative splicing [38]. GPCR isoforms differ in their subcellular locations, signaling pathways, post-transcriptional regulation and their physiological functions [39, 40]. Moreover, in specific tissues, such as airway smooth muscle, GPCRs are expressed extensively and are frequently alternative spliced to create a highly diversified receptor milieu [41]. Although identification and characterization of alternative spliced genes within GPCR superfamily has not been delineated, it is widely accepted that AS is common in this family and influenced by changing patho-physiological conditions [38, 42]. In *S*. *japonicum*, we identified 71 members of the GPCR family, 31 of which were comprised of one exon, and 12 of remaining 40 multi-exon members had undergone AS. Given that different life stages may experience different stimuli in hosts, some GPCR genes may undergo retrogressive evolution during the complex parasitic lifecycle. The discrepancy in the number of GPCR genes that exhibited AS in humans and in *S*. *japonicum* is likely a consequence of different evolutionary history of these two species.

In contrast, we found high rates of AS in TGF-β gene family. TGF-β is a secreted cytokine that binds to high-affinity serine/threonine kinase receptors and transduces intracellular signals via Smad proteins. TGF-β superfamily is comprised of TGF-β, bone morphogenetic proteins, activins and growth and differentiation factors. These proteins are ubiquitous in multicellular organisms and are involved in intercellular signaling [43]. There are many reports of AS in TGFs, member receptors and other members of this superfamily, and it is known that TGF regulates AS in other genes [44–48]. As shistosomes have complex lifecycle and adults are parasitic, intercellular signaling may differ among different life stages and thus, requires different isoforms of TGF-β and/or their adaptors and effectors. As parasites, the multicellular adult worm would sense its host’s immune system and respond by becoming “invisible” to the host. This host-parasite interaction makes intercellular signaling very important. Thus, genes of signal proteins TGF-β and related proteins may undergo more AS events to produce different isoforms among different life stages, especially when some are in parasitic stages.

High rate of AS events we observed in SWI/SNF gene family are easily understood in terms of their roles in nucleus. This gene family functions as a chromatin remodeling complex, and use energy from ATP hydrolysis to alter the structure of nucleus and activate gene expression [49]. To date, few reports have identified AS events in this gene family [50, 51]. Based on our results, we suggest that during the complex lifecycle of this parasitic helminth, SWI/SNF complexes mediate expression of different genes to cope with different environments. Alternative variants of SWI/SNF family would increase variability and allow better modulation of this process of gene activation or inactivation.

In our attempt to search for alternative splice motifs in *S*. *japonicum*, we failed to find any motif. In fact, no featured alternative splicing motif has been characterized in numerous species. Hence, it seems that AS process and allocation of exons and introns may rely more on spliceosome recognition and interaction with RNA and proteins than on sequences [52]. There must be other mechanisms by which the spliceosome recognizes alternative or constitutive exons and introns. Although no motif has been found, our analysis did reveal primary donor site GT (~90%) and acceptor site AG (~90%). This is identical to high eukaryotes and model organisms, as across diverse phyla intron boundary has been revealed to be conserved to GT-AG rule, with very low GC-AG events[26]. Studies has reported that while U2-type spliceosome processes GT–AG and GC–AG introns in pre-mRNAs, U12-type processes the minor AT–AC and the so-called U12-type GT–AG introns in metazoan [53]. Also, U2-type introns are found to undergo more AS events than U12-type introns [54]. Nevertheless, this comformity in the parasite and its mammal hosts may illustrate same patterns in gene expression and regulation. This regulates the complex lifecycle of *S*. *japonicum* with precise control of its physiological processes.

Secretory and excretory proteins of parasites play vital roles in host-worm interactions, and modulate host’s immune response during infection to mediate the helminth’s survival inside the host [55]. In this respect, some transmembrane proteins are interspersed in the tegument to imitate host self-antigens to evade the immune system. If these proteins result from AS events, their counterparts from the same gene will be of great interest as a means of characterizing their functions in the secretory, excretory or intracellular compartments. Therefore, genes encoding secretory and transmembrane proteins that undergo AS will provide scope for elucidating information about interactions between parasites and their hosts. These proteins are also attractive candidate targets for therapeutic use. However, these results were derived from bioinformatics analysis and predictions, and further experimental studies are needed to verify the results.

In conclusion, with the support of next-generation sequencing technology and bioinformatics tools, we were able to analyze global transcriptome among adult and schistosomula stages of *S*. *japonicum*. We aimed at searching alternative splicing events in the transcriptome analyzed. We discovered 11,623 AS events in 7,099 genes in these two life stages. The average proportion of AS events per gene was 42.14%. The AS rate for some gene families such as SWI/SNF family and TGF-β family are much higher than average. Alternative variants of the families would increase variability and allow better modulation of the process of gene activation or inactivation. In addition, we found that exon skip is the most common type of alternative splicing events in schistosome as founded in high eukaryotes, while intron retention is the least common alternative splicing type. This is close to its mammal hosts and may indicate close relationship between the parasite and its hosts. In terms of secretory and excretory protein encoding genes, alternative splicing events occurred in these genes made it interesting about how transcriptional modifications regulate proteins intracellular or extracellular locations and modify functions of the proteins. Our study provides more insights into genome and transcriptome sequences and more information into the biology of the parasite.

## Supporting Information

S1 Fig Venn diagrams for known genes (A) and novel genes (B) predicted in both studies. (TIF)

S1 Table Information on sequencing sample and re-mapping of reads to genome in this study. (TIF)

S2 Table Gene expression profile in adult and schistosomulum stage of *Schistosoma japonicum*. (XLS)

S3 Table PCR primers used in AS identification. (XLSX)

S4 Table Comparison of the data from two studies. (TIF)

S5 Table List of overlapping known genes identified in this study. (XLSX)

S6 Table List of unique known genes identified in this study. (XLSX)

S7 Table List of overlapping novel genes identified in this study. (XLSX)

S8 Table List of novel genes unique in this study. (XLSX)
