# Supplementary figures and images for: Transcriptome Bioinformatical Analysis of Vertebrate Stages of Schistosoma japonicum Reveals Alternative Splicing Events
Source: PLoS One. 2015 Sep 25;10(9):e0138470. doi: 10.1371/journal.pone.0138470 (PMC4583307; doi:10.1371/journal.pone.0138470)

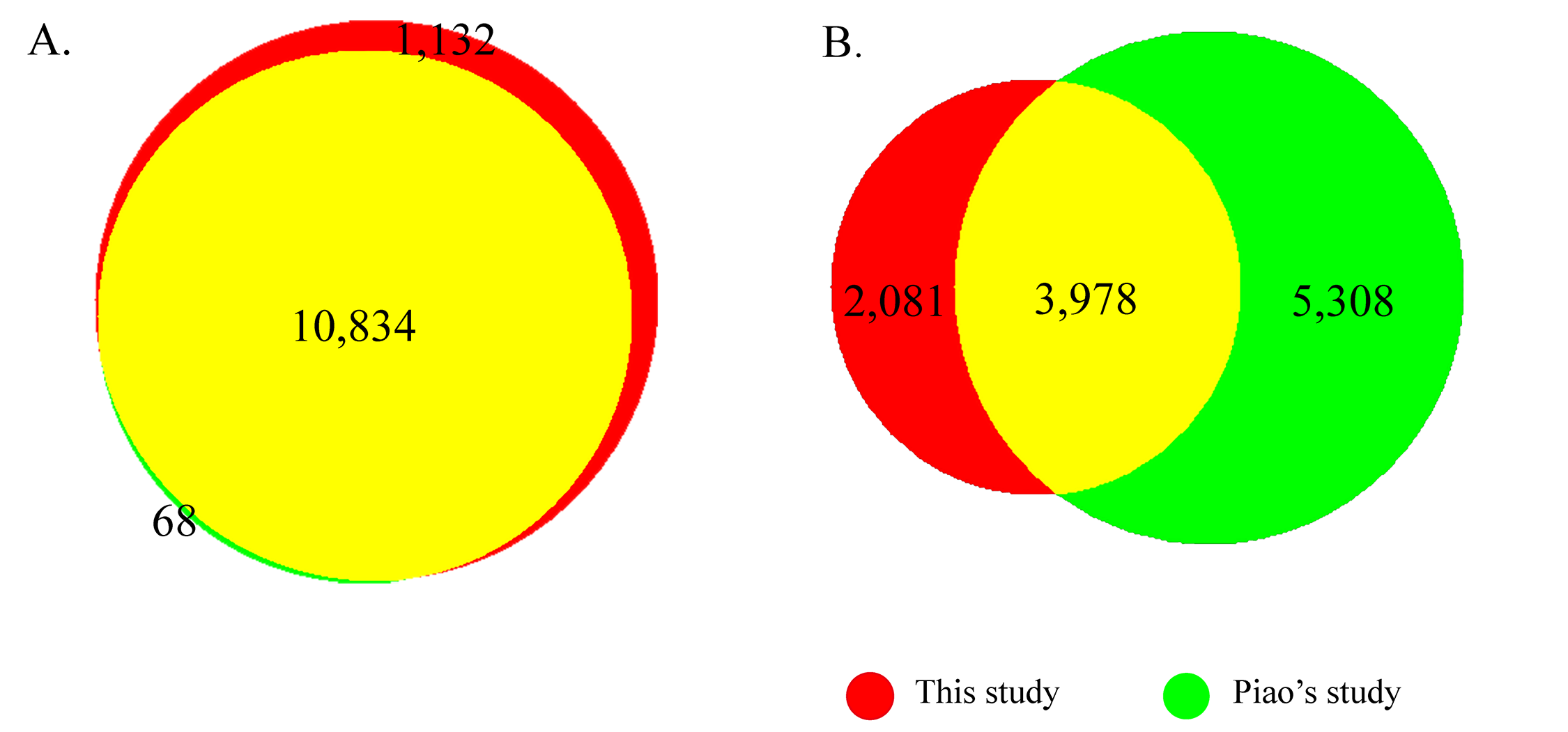

Supplement: S1 Fig — (TIF) [file pone.0138470.s001.tif]

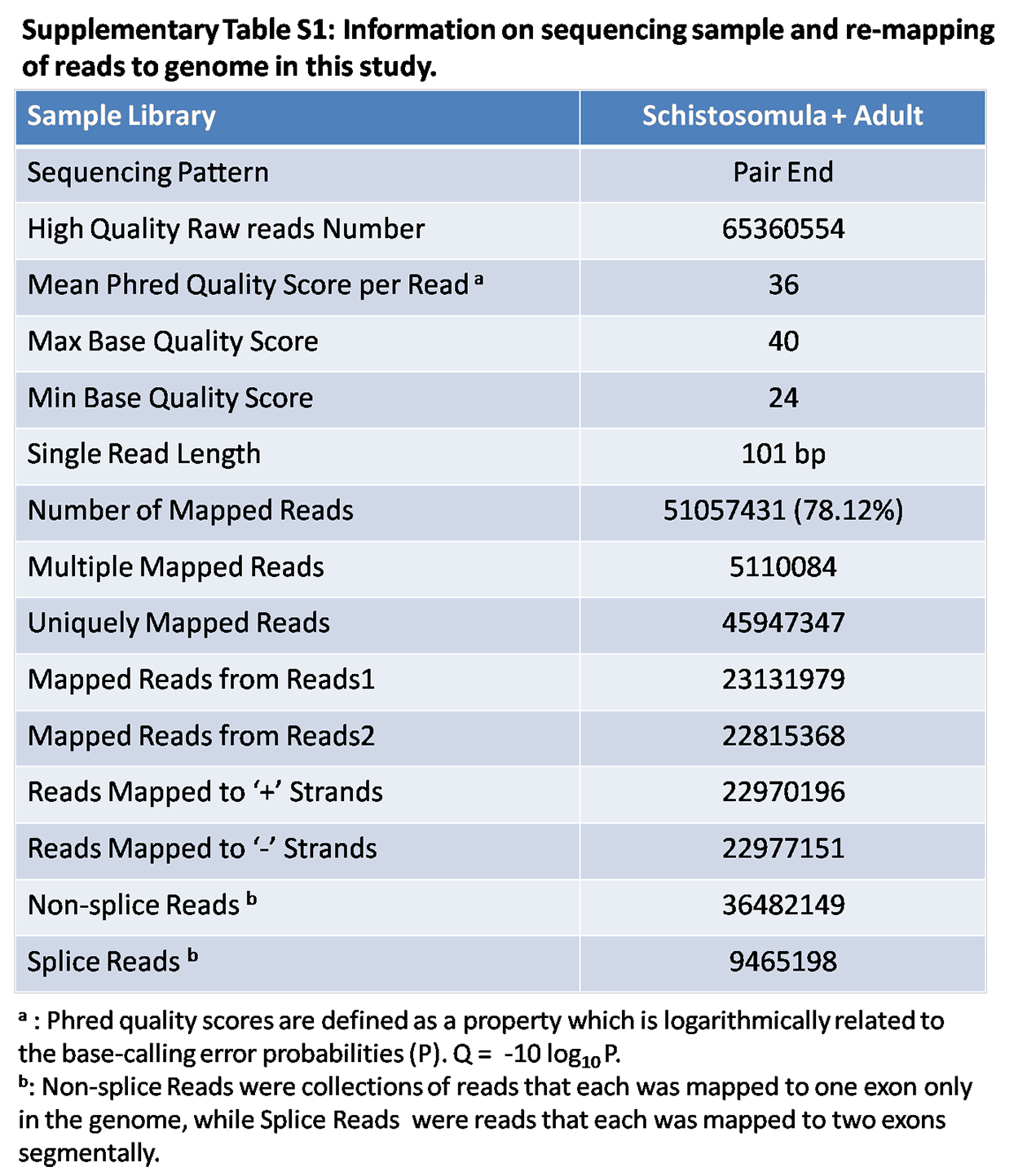

Supplement: S1 Table — (TIF) [file pone.0138470.s002.tif]
